# Tau Protein Accumulation Trajectory-Based Brain Age Prediction in the Alzheimer’s Disease Continuum

**DOI:** 10.3390/brainsci14060575

**Published:** 2024-06-04

**Authors:** Min Wang, Min Wei, Luyao Wang, Jun Song, Axel Rominger, Kuangyu Shi, Jiehui Jiang

**Affiliations:** 1 School of Life Sciences, Shanghai University, Shanghai 200444, China; 2 Department of Neurology, XuanWu Hospital of Capital Medical University, Beijing 100053, China; 3 Department of Nuclear Medicine, Inselspital, Bern University Hospital, University of Bern, 3010 Bern, Switzerland; 4 Computer Aided Medical Procedures, School of Computation, Information and Technology, Technical University of Munich, 85748 Munich, Germany

**Keywords:** brain age, Alzheimer’s disease, tau protein accumulation, positron emission tomography

## Abstract

Clinical cognitive advancement within the Alzheimer’s disease (AD) continuum is intimately connected with sustained accumulation of tau protein pathology. The biological brain age and its gap show great potential for pathological risk and disease severity. In the present study, we applied multivariable linear support vector regression to train a normative brain age prediction model using tau brain images. We further assessed the predicted biological brain age and its gap for patients within the AD continuum. In the AD continuum, evaluated pathologic tau binding was found in the inferior temporal, parietal-temporal junction, precuneus/posterior cingulate, dorsal frontal, occipital, and inferior-medial temporal cortices. The biological brain age gaps of patients within the AD continuum were notably higher than those of the normal controls (*p* < 0.0001). Significant positive correlations were observed between the brain age gap and global tau protein accumulation levels for mild cognitive impairment (*r* = 0.726, *p* < 0.001), AD (*r* = 0.845, *p* < 0.001), and AD continuum (*r* = 0.797, *p* < 0.001). The pathologic tau-based age gap was significantly linked to neuropsychological scores. The proposed pathologic tau-based biological brain age model could track the tau protein accumulation trajectory of cognitive impairment and further provide a comprehensive quantification index for the tau accumulation risk.

## 1. Introduction

Alzheimer’s disease (AD) is a prevalent neurodegenerative disease characterized by a typically long preclinical stage lasting 15–20 years [1]. The 2018 National Institute on Aging and Alzheimer’s Association research framework primarily characterizes AD by the accumulation of β-amyloid (Aβ) and tau pathologic proteins and subtle neurodegeneration [AT(N)] [2]. Previous studies have demonstrated that Aβ accumulation may play a causal upstream role in the AD continuum, potentially leading to the downstream pathologic changes, such as tauopathy and neurodegeneration, that ultimately result in cognitive deterioration [3,4]. However, the potential floor or ceiling effects in the amount of Aβ deposition may have limited contributions to the degree of dementia [5,6]. The ongoing tau pathologic accumulation, rather than Aβ alone, closely corresponds with the clinical progression and cognitive changes of AD [7,8,9,10,11]. Thus, characterizing the tau protein accumulation trajectory is of pivotal clinical importance for determining the severity of AD.

Thanks to advancements in positron emission tomography (PET) imaging and traces, tau PET imaging enables in vivo visualization and quantification of the AD-related tau protein accumulation trajectory. Currently, semi-quantitative methods like standardized uptake value ratio (SUVR) are primarily utilized in clinical practice to assess tau protein accumulation. However, the heterogeneity and individual specificity of AD make it challenging to provide patients with a relatively simple and easy-to-understand quantitative parameter. Recent studies have proposed a superior canonical image-based quantitative approach, tau^IQ^, to quantify tau PET scans. This approach exhibits enhanced efficacy compared to traditional SUVR approaches [12,13]. However, the approach necessitates a large cross-sectional dataset of subjects across all of the AD continuum to estimate nonspecific tau load, local tau load, and noise, and these data requirements restrict its application in clinical practice [12].

Recent studies have adopted approaches based on biological brain aging to assess disease-specific risk and elucidate the determinants contributing to the discrepancy between biological brain age and chronological age [14,15,16,17]. In particular, the efficacy of predictive models for estimating brain age using neuroimaging, such as magnetic resonance imaging (MRI) and fluorodeoxyglucose PET, has been demonstrated in AD, Parkinson’s disease, epilepsy, and schizophrenia [18,19,20,21,22]. The biological brain age derived from a brain image is typically related to the reduction of gray matter, decreased glucose metabolism, cerebral blood flow, or global oxygen utilization [23,24,25]. The discrepancy between chronological brain age and biological brain age, termed the brain age gap, can provide insight into whether a patient within the AD continuum appears older or younger compared to a same-aged individual with normal cognitive status. Accumulating evidence from AD studies suggests that tau pathology initially manifests in the locus coeruleus and entorhinal cortex [26]. Very few studies, however, have examined biological brain age using pathologic tau accumulation in AD spectrum [27]. Moreover, biological brain age and its gap can capture individual tau accumulation differences in the interaction of aging and AD patients.

In the current observational study, our objective was to develop a normative brain age prediction model using AV-1451 tau PET images of normal controls (NCs) and further assess the predicted brain age and its gap for patients within the AD continuum, including mild cognitive impairment (MCI) and AD. We also examined the associations between brain age gap and neuropsychological tests for patients with cognitive impairment.

## 2. Materials and Methods

### 2.1. Participants

All of the imaging data utilized in this study were acquired from the Alzheimer’s Disease Neuroimaging Initiative (ADNI) database (https://adni.loni.usc.edu/, accessed on 31 March 2005). The ADNI was launched in 2003 as a public-private partnership, led by Principal Investigator Michael W. Weiner, MD. The primary goal of ADNI has been to test whether serial MRI, PET, other biological markers, and clinical and neuropsychological assessments can be combined to measure the progression of MCI and early AD. All procedures performed in this study involving human participants were in accordance with the ethical standards of the institutional and/or national research committee and with the 1964 Declaration of Helsinki and its later amendments or comparable ethical standards. The data analysis and ethical permissions of this study were approved by the institutional review board at each of the participating centers. ADNI is listed in the ClinicalTrials.gov registry (ADNI-1: NCT00106899, date: 31 March 2005; ADNI-GO: NCT01078636, date: 1 March 2010; ADNI-2: NCT0123197, date: 27 October 2010; ADNI-3: NCT02854033, date: 27 July 2016).

A total of 810 subjects (418 NCs, 306 patients with MCI, and 86 patients with AD) were included in this study. The T1-weighted MRI and AV1451 PET images of all subjects had to be obtained within the same visit. Mini-Mental State Examination (MMSE), Montreal Cognitive Assessment (MOCA), Alzheimer’s Disease Assessment Scale Cognitive 11 items (ADAS11), and Alzheimer’s Disease Assessment Scale Cognitive 13 items (ADAS13) were used to evaluate the cognitive function in all participants. All of the patients diagnosed with AD met the diagnostic criteria outlined by the National Institute of Neurological and Communicative Disorders and Stroke and the Alzheimer’s Disease and Related Disorders Association [28]. Detailed diagnostic criteria for all subjects are available at https://adni.loni.usc.edu/methods/documents/ (accessed on 31 March 2005).

### 2.2. Image Acquisition and Processing

We obtained the T1-weighted MRI and corresponding AV1451 PET images from the ADNI database. Details on the acquisition and preprocessing procedures for T1 and PET images can be accessed (http://adni.loni.usc.edu/methods/documents/, accessed on 31 March 2005). In brief, tau PET images were acquired during a resting state 75–105 min after an intravenous bolus injection of ^18^F-radiolabeled AV1451. T1 MRI were obtained using unified scanning protocols on 3T scanners.

Individual T1 MRI images were segmented using Statistical Parametric Mapping 12 (SPM12) carried out in MATLAB 2021b (MathWorks, Natick, MA, USA). Tau PET images were realigned, averaged, and spatially coregistered with their corresponding MRI images. Subsequently, voxel-based partial volume effects correction of tau PET images was performed using the Müller-Gärtner method with the PETPVE12 toolbox. Then, the AV1451 PET images were normalized to the Montreal Neurological Institute standard space via applying the MRI-segmented parameters. Spatially normalized PET images were subsequently smoothed using a Gaussian kernel with 8 mm full-width at half-maximum. The SUVR image was generated using the inferior cerebellar gray matter as the reference region. We extracted 80 cerebral averaged SUVR values from the preprocessed PET images utilizing the automated anatomical labeling atlas. We also calculated a global merged SUVR from the typical temporal meta-region harboring elevated pathologic tau accumulation in the AD continuum (inferior temporal, the middle temporal and fusiform gyri, the parahippocampal, the entorhinal cortex) [29].

### 2.3. Brain Age Estimation

We used the resulting 80 cortical tau SUVR values as features to estimate the values to be used in the normative brain age predictive model. A multivariable linear support vector regression (SVR) model was initially trained to predict individual chronological age utilizing these tau SUVR values. Compared to conventional linear regression methods, the SVR model offers enhanced robustness against outliers and overfitting by learning the relative importance of each SUVR value in age prediction and fitting a hyperplane to the brain age. We employed a 10-fold cross-validation iteration approach to train and predict chronological age utilizing NCs consisting of 9 folds (training dataset, *n* = 376 [418 × 0.9]). The fitted regression coefficients in the SVR model were subsequently applied iteratively to the held-out set of individuals (test dataset, *n* = 42), which resulted in a prediction of chronological age for every NC participant. The SVR model employed sequential minimal optimization for solving for the chronological age, with a set gap tolerance of 0.001.

The brain age gap offers a standardized measurement indicating that an individual’s brain tau accumulation level appeared older (gap > 0) or younger (gap < 0) compared to same-aged NCs without cognitive impairment. The brain age gap correlates with chronological age, leading to an overestimation for younger participants and an underestimation for older attributed to regression dilution [30,31,32]. Consequently, we utilized the linear bias correction method to address age bias correction for the brain age gap, where sex, education, and APOE4 status were adjusted. The trained SVR model and the corrected brain age gap model were then applied to patients with MCI and AD to obtain pathologic tau accumulation-based brain age and its gap estimates for each cohort. The accuracy of the predictions of the brain age of NCs was evaluated using mean absolute error (MAE) and Spearman’s correlation coefficient between chronological age and predicted brain age in the test dataset.

### 2.4. Statistical Analyses

All continuous data underwent normality testing using the Kolmogorov–Smirnov test, and the homogeneity of variance was determined using the F test. Group differences among NCs, MCI, and AD in clinical characteristics and brain age gap were evaluated using one-way analysis of variance (ANOVA) with post hoc Bonferroni’s correction and pairwise comparisons. The effect size was used to measure the pairwise comparisons. Power analysis for ANOVA was conducted in G-Power to determine a sufficient sample size using an alpha of 0.05, a power of 0.95, a large effect size (*f* = 0.4), a number of groups of 3, and two tails. Based on the aforementioned assumptions, the desired sample size is 102. A chi-squared test was used to analyze the sex variable. To investigate the associations between brain age gap and neuropsychological assessments, Spearman’s correlation coefficients were calculated. Additionally, we repeated the above association assessments using covariate controlled (sex, education, APOE4 status) linear regression models, to ensure that the relationship between brain age gap and neuropsychological assessments was not driven by these covariates. The Spearman’s correlation coefficient was employed to assess the association of the brain age gap and the merged tau SUVR. All statistical analyses were conducted with MATLAB 2021b (MathWorks) and Prism v. 10.1.2 (GraphPad Software). Significance was determined at *p* < 0.05 (two-tailed).

## 3. Results

### 3.1. Subject Characteristics

The clinical and demographic information for this study is shown as Table 1. Compared with AD, NC and patients with MCI had younger age and higher education. There was no significant sex difference (*p* = 0.203). Compared with NC, patients with MCI and AD had higher cognitive severity (ADAS11, ADAS13, MMSE, and MOCA score) and evaluated pathologic tau accumulation (all *p* < 0.001). Average chronological age (range) across NC, MCI, and AD was 72.7 (52.7 to 92.5), 74.6 (55.9 to 92.3), and 76.9 (55.3 to 91.1), respectively.

### 3.2. Pathologic Tau Accumulation in AD Continuum

**Figure 1:**
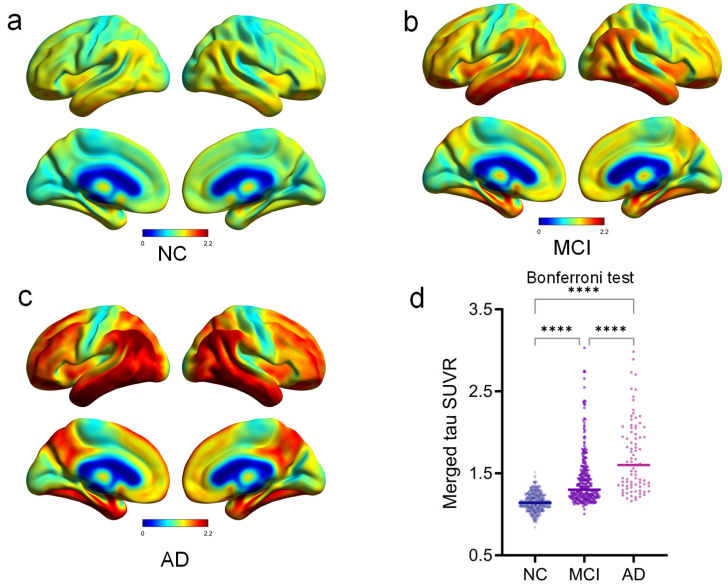
Group-average tau PET SUVR maps. Compared with NC group (**a**), there are significant and widespread pathologic tau accumulations in the MCI (**b**) and AD (**c**) groups. (**d**) Group differences of merged tau SUVR between NC, MCI, and AD. NC: normal control, MCI: mild cognitive impairment, AD: Alzheimer’s disease, SUVR: standardized uptake value ratio, ****: Post hoc *p* < 0.0001.

As shown in Figure 1, across the AD continuum, the evaluated pathologic tau binding was maximal at the inferior temporal, parietal-temporal junction, and precuneus/posterior cingulate and moderately spread in the dorsal frontal, occipital, and inferior-medial temporal cortices. Group differences of global tau accumulation also show that AD had the highest tau deposition levels, SUVR = 1.71 ± 0.63 (post hoc *p* < 0.0001, Cohen’s *d* = 1.73 between NC and AD; Cohen’s *d* = 0.72 between MCI and AD, Figure 1d).

### 3.3. Brain Age Prediction and Its Gap

**Figure 2:**
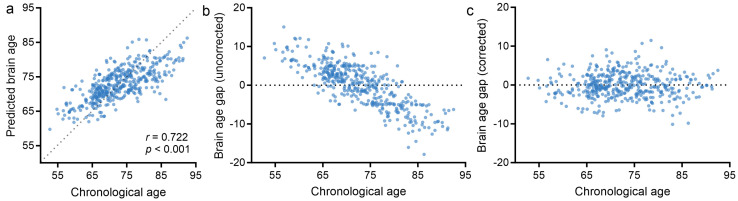
Brain age prediction on normal controls. (**a**) Regression plot showing chronological age versus predicted brain age; (**b**) Uncorrected brain age gap; (**c**) Brain age gap after bias correction.

The normative brain age prediction model was trained using tau PET images with 10-fold cross-validation. As depicted in Figure 2a, the overall accuracy measured on the test dataset was MAE = 4.89 ± 0.261, *r* = 0.722, *p* < 0.001. Figure 2b,c demonstrate the scatter plot of chronological age and predicted brain age gap. After linear bias correction, there was no significant negative correlation between chronological age and predicted gap.

**Figure 3:**
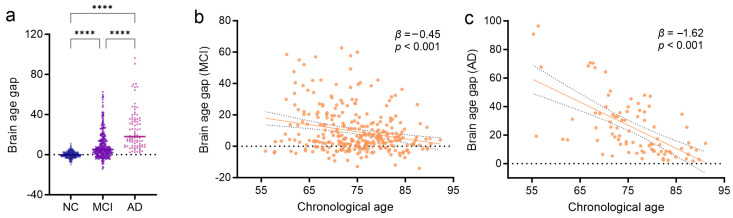
Pathologic tau-based brain age gap estimation for MCI and AD groups. (**a**) Violin plots of the corrected brain age gap for each diagnostic group. The corrected brain age gap of disease groups was compared with cognitively unimpaired individuals using a one-way ANOVA with post hoc Bonferroni’s correction. Tau-based brain age gap estimation for MCI (**b**) and AD (**c**). NC: normal control, MCI: mild cognitive impairment, AD: Alzheimer’s disease, ****: Post hoc *p* < 0.0001.

We further calculated the biological brain age and its gap in patients with MCI and AD utilizing the normative trained SVR prediction model and AV1451 PET images. As anticipated, the brain age gaps of the cognitive impairment groups were significantly higher than that of the NCs (*p* < 0.0001, multiple comparisons *p* < 0.0001, Figure 3). The mean brain age gap of the MCI/AD was 9.41 ± 13.8 or 25.4 ± 20.7 (Cohen’s *d* = 1.71 between NC and AD; Cohen’s *d* = 0.94 between NC and MCI; Cohen’s *d* = 0.90 between MCI and AD). We also observed that there was significant negative association between chronological age and brain age gap (MCI: *β* = −0.45, *p* < 0.001, *R^2^* = 0.055; AD: *β* = −1.62, *p* < 0.001, *R^2^* = 0.38), which indicated that patients with younger disease onset tended to have higher brain age gaps derived from pathologic tau accumulation.

### 3.4. Associations between Brain Age Gap and Neuropsychological Assessments and the AD Biomarker

**Figure 4:**
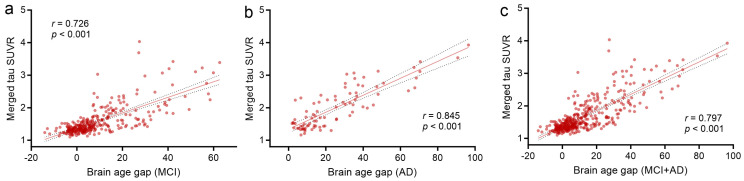
Association of brain age gap with merged tau SUVR in AD continuum. Scatter plots show the relationship between tau-based brain age gap with merged tau PET SUVR for MCI (**a**), AD (**b**), the whole AD continuum (**c**), respectively. MCI: mild cognitive impairment, AD: Alzheimer’s disease; SUVR: standardized uptake value ratio.

We further investigated the associations between brain age gap estimated by tau PET images and neuropsychological assessments and the AD biomarker, to evaluate whether a higher brain age gap was associated with severe cognitive symptoms. As shown in Figure 4, we found there were notable positive correlations between brain age gap and global tau accumulation level for MCI (*r* = 0.726, *p* < 0.001), AD (*r* = 0.845, *p* < 0.001), and the whole AD continuum (*r* = 0.797, *p* < 0.001), which remained after controlling for covariates (MCI: *β* = 0.024, *p* < 0.001, *R^2^* = 0.53; AD: *β* = 0.026, *p* < 0.001, *R^2^* = 0.71; whole AD continuum: *β* = 0.025, *p* < 0.001, *R^2^* = 0.63).

**Figure 5:**
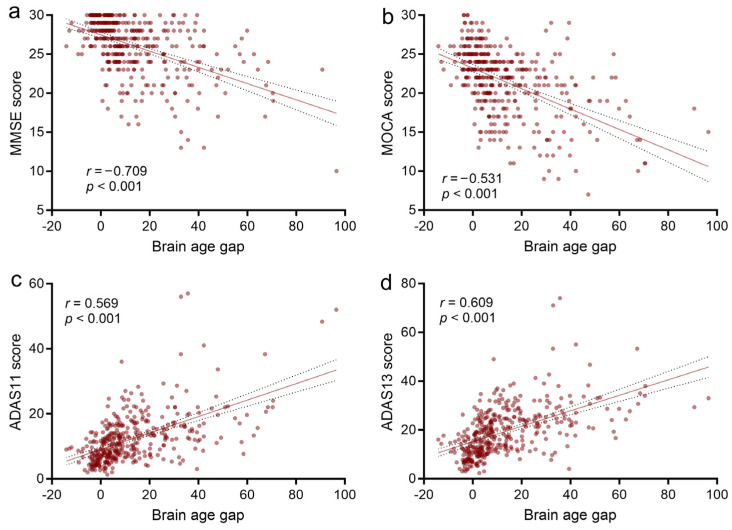
Association of brain age gap with neuropsychological assessments in the AD continuum. Scatter plots show the relationship between tau-based brain age gap with MMSE score (**a**), MOCA score (**b**), ADAS11 score (**c**), and ADAS13 score (**d**), respectively. ADAS11, Alzheimer’s Disease Assessment Scale Cognitive 11 items; ADAS13, Alzheimer’s Disease Assessment Scale Cognitive 13 items; MMSE, mini-mental status exam; MOCA, Montreal Cognitive Assessment.

As expected, the pathologic tau-based brain age gap was significantly associated with MMSE score (*r* = −0.709, *p* < 0.001), MOCA score (*r* = −0.531, *p* < 0.001), ADAS11 score (*r* = 0.569, *p* < 0.001), and ADAS13 score (*r* = 0.609, *p* < 0.001) in AD continuum (MCI and AD groups, Figure 5). After adjustments for sex, education, and APOE4 status, there were similar significant associations between the brain age gap and MMSE score (*β* = −0.104, *p* < 0.001, *R^2^* = 0.24), MOCA score (*β* = −0.131, *p* < 0.001, *R^2^* = 0.25), ADAS11 score (*β* = 0.253, *p* < 0.001, *R^2^* = 0.31), and ADAS13 score (*β* = 0.317, *p* < 0.001. *R^2^* = 0.29).

## 4. Discussion

Given the severe and irreversible nature of AD, it is crucial to precisely explore the pathology trajectory within the AD continuum. The tau protein pathology, as an essential biomarker for diagnosis and cognitive progression, exhibits successive spatial expansions during the course of AD. In light of that, our study presents an optimized brain age gap prediction model which is based on the tau accumulation trajectory. The brain age gaps of the cognitive impairment groups were significantly higher than that of the NCs, with a negative correlation observed between chronological age and brain age gap. In participants with cognitive impairment, the brain age gap exceeded that of cognitively unimpaired participants and exhibited a notable correlation with both neuropsychological score and neuroimaging biomarkers. This study presents an optimal model for predicting brain age and the brain age gap in individuals within the AD continuum, emphasizing the significance of the brain age gap marker and its potential as a valuable tool for identifying individuals at risk. Furthermore, it may serve as a valuable biomarker to detect heightened risk for tau pathology or indicate disease progression.

In the past, there have been numerous studies regarding brain aging. Most of these studies have predominantly employed structural MRI and fluorodeoxyglucose PET imaging for estimations of aging [31,33,34,35]. However, tau PET exhibits greater sensitivity compared to amyloid-PET and cortical thickness measurements in exploring cognitive variations during the early stages of AD [36]. Our model accurately estimated the disparity an individual’s brain age gap using pathologic tau images. One finding that stands out is that the evaluated pathologic tau binding was maximal at the inferior temporal, parietal-temporal junction, and precuneus/posterior cingulate and moderately spread in the dorsal frontal, occipital, and inferior-medial temporal cortices in AD continuum, which is in line with previous research to some extent [37,38]. Imaging analysis revealed crucial pathologic tau regions within the AD continuum and demonstrated significant differences in overall tau protein accumulation. The studies conducted in vitro have indicated that tau proteins might experience transneuronal spreading, and estimating normal values for these brain regions was crucial to our development of the model used in this study [39].

The brain age gap derived from the tau PET data is elevated in cognitively impaired individuals compared to NCs. This performance is slightly better than previous structural age prediction models, which show increases between five and ten years [40]. Meanwhile, this model is clearly sensitive to different groups representing various statuses in the symptomatic phase. In cognitively impaired participants, the brain age gap estimated from model was significantly associated with the global tau accumulation level, further demonstrating the model’s predictive capability for tau pathology deposition. Elevated tau protein deposits were closely linked to cognitive deficits in AD [41,42]. Therefore, the brain age gap estimated by our model can systematically assess tau protein deposition and cognitive impairment in the brain, providing a straightforward and comprehensive index for clinical use. This could assist in early clinical intervention and help delay further cognitive decline. Additionally, this brain age gap model has great potential for initiating physical examinations in aging individuals and providing early cognitive risk predictions.

Interestingly, in MCI and AD, chronological age showed a negative correlation with the brain age gap, revealing individuals with earlier disease onset typically exhibit a higher brain age gap resulting from pathological tau accumulation. A larger disparity between brain age and chronological age signifies a heightened risk during the course of the disease [43]. Pathologic tau-based brain age gaps demonstrated a significant association with neuropsychological scores, underscoring the intricate relationship between patterns of tau accumulation and cognitive function with clinical symptoms [44].

In our current study, various limitations need to be addressed. Firstly, this is a cross-sectional study, and future follow-up is necessary to further validate the effectiveness of model. Secondly, in consideration of the potential influence of cross-cultural backgrounds, this study did not include results from multiple cohorts. Our future plans include expanding multicenter research to obtain more stable models. Thirdly, the training set might have a smaller sample size compared to previous models. In addition, other types of biomarkers, such as plasma and cerebrospinal fluid markers [45], need to be incorporated to complement the ATN framework and achieve a more complete model in the future. Given the previous research on brain age [46,47,48], we believe this model could be utilized as a sensitive biomarker for cognitive function decline during the symptomatic stage.

## 5. Conclusions

In summary, the current study demonstrates a brain age prediction model which was developed using the tau accumulation trajectory of normal controls. The model generates accurate brain age and brain age gap predictions for cognitively impaired individuals in the AD continuum. In the future, as effective treatments for AD become available, the model might assist in identifying individuals at various stages. Overall, all of our findings provide insight into the pathophysiology of AD and the potential predictive ability of our model to enable personalized recognition.

## Figures and Tables

**Table 1:** Clinical and demographic information about this study subject.

	NC	MCI	AD	*p*	Post Hoc *p*		
					*p* ^1^	*p* ^2^	*p* ^3^
Number	418	306	86	-	-	-	-
Sex (M/F)	259/159	170/136	54/32	0.203	0.083	0.885	0.232
Age (years)	72.7 ± 7.6	74.6 ± 7.3	76.9 ± 7.9	<0.001	0.0014	<0.001	0.341
Education (years)	16.7 ± 2.2	16.4 ± 2.5	15.4 ± 2.5	<0.001	0.231	<0.001	0.003
APOE4 carriers (%)	32.3	53.9	61.6	-	-	-	-
ADAS11	5.2 ± 2.5	9.7 ± 4.4	20.6 ± 8.6	<0.001	<0.001	<0.001	<0.001
ADAS13	8.2 ± 3.9	15.8 ± 6.8	31.5 ± 10.1	<0.001	<0.001	<0.001	<0.001
MMSE	29.2 ± 1.0	27.3 ± 2.3	21.5 ± 4.2	<0.001	<0.001	<0.001	<0.001
MOCA	26.2 ± 2.6	22.9 ± 3.2	16.3 ± 4.4	<0.001	<0.001	<0.001	<0.001
Merged tau SUVR	1.14 ± 0.12	1.42 ± 0.45	1.71 ± 0.63	<0.001	<0.001	<0.001	<0.001

Data are expressed as means ± standard deviations or rate. Post hoc *p* values were calculated after application of the Bonferroni’s correction. *p*^1^ NC versus patients with MCI. *p*^2^ NC versus patients with AD. *p*^3^ patients with MCI versus patients with AD. Abbreviations: NC, normal control; MCI: mild cognitive impairment; AD, Alzheimer’s disease; ADAS11, Alzheimer’s Disease Assessment Scale Cognitive 11 items; ADAS13, Alzheimer’s Disease Assessment Scale Cognitive 13 items; MMSE, mini-mental status exam; MOCA, Montreal Cognitive Assessment; SUVR, standardized uptake value ratio.

## Data Availability

The data supporting the findings of this study are openly available in the Alzheimer’s Disease Neuroimaging Initiative at https://adni.loni.usc.edu/, accessed on 31 March 2005.

## References

[1] (2023). 2023 Alzheimer’s Disease Facts and Figures. Alzheimers Dement..

[2] Jack C.R., Bennett D.A., Blennow K., Carrillo M.C., Dunn B., Haeberlein S.B., Holtzman D.M., Jagust W., Jessen F., Karlawish J. (2018). NIA-AA Research Framework: Toward a Biological Definition of Alzheimer’s Disease. Alzheimers Dement..

[3] Jack C.R., Holtzman D.M. (2013). Biomarker Modeling of Alzheimer’s Disease. Neuron.

[4] Jansen W.J., Ossenkoppele R., Knol D.L., Tijms B.M., Scheltens P., Verhey F.R., Visser P.J., Aalten P., Aarsland D., Alcolea D. (2015). Prevalence of Cerebral Amyloid Pathology in Persons without Dementia: A Meta-Analysis. JAMA.

[5] Murphy M.P., LeVine H. (2010). Alzheimer’s Disease and the Amyloid-Beta Peptide. J. Alzheimers Dis..

[6] Thal D.R., Ronisz A., Tousseyn T., Rijal Upadhaya A., Balakrishnan K., Vandenberghe R., Vandenbulcke M., von Arnim C.A.F., Otto M., Beach T.G. (2019). Different Aspects of Alzheimer’s Disease-Related Amyloid β-Peptide Pathology and Their Relationship to Amyloid Positron Emission Tomography Imaging and Dementia. Acta Neuropathol. Commun..

[7] Vogel J.W., Young A.L., Oxtoby N.P., Smith R., Ossenkoppele R., Strandberg O.T., La Joie R., Aksman L.M., Grothe M.J., Iturria-Medina Y. (2021). Four Distinct Trajectories of Tau Deposition Identified in Alzheimer’s Disease. Nat. Med..

[8] Strikwerda-Brown C., Hobbs D.A., Gonneaud J., St-Onge F., Binette A.P., Ozlen H., Provost K., Soucy J.-P., Buckley R.F., Benzinger T.L.S. (2022). Association of Elevated Amyloid and Tau Positron Emission Tomography Signal with Near-Term Development of Alzheimer Disease Symptoms in Older Adults without Cognitive Impairment. JAMA Neurol..

[9] Ossenkoppele R., Pichet Binette A., Groot C., Smith R., Strandberg O., Palmqvist S., Stomrud E., Tideman P., Ohlsson T., Jögi J. (2022). Amyloid and Tau PET-Positive Cognitively Unimpaired Individuals Are at High Risk for Future Cognitive Decline. Nat. Med..

[10] Biel D., Brendel M., Rubinski A., Buerger K., Janowitz D., Dichgans M., Franzmeier N. (2021). Alzheimer’s Disease Neuroimaging Initiative (ADNI) Tau-PET and in Vivo Braak-Staging as Prognostic Markers of Future Cognitive Decline in Cognitively Normal to Demented Individuals. Alzheimers Res. Ther..

[11] Pontecorvo M.J., Devous M.D., Kennedy I., Navitsky M., Lu M., Galante N., Salloway S., Doraiswamy P.M., Southekal S., Arora A.K. (2019). A Multicentre Longitudinal Study of Flortaucipir (18F) in Normal Ageing, Mild Cognitive Impairment and Alzheimer’s Disease Dementia. Brain.

[12] Whittington A., Gunn R.N. (2021). Alzheimer’s Disease Neuroimaging Initiative TauIQ: A Canonical Image Based Algorithm to Quantify Tau PET Scans. J. Nucl. Med..

[13] Whittington A., Gunn R.N. (2019). Amyloid Load: A More Sensitive Biomarker for Amyloid Imaging. J. Nucl. Med. Off. Publ. Soc. Nucl. Med..

[14] Tian Y.E., Cropley V., Maier A.B., Lautenschlager N.T., Breakspear M., Zalesky A. (2023). Heterogeneous Aging across Multiple Organ Systems and Prediction of Chronic Disease and Mortality. Nat. Med..

[15] Beheshti I., Ganaie M.A., Paliwal V., Rastogi A., Razzak I., Tanveer M. (2022). Predicting Brain Age Using Machine Learning Algorithms: A Comprehensive Evaluation. IEEE J. Biomed. Health Inform..

[16] Han L.K.M., Dinga R., Hahn T., Ching C.R.K., Eyler L.T., Aftanas L., Aghajani M., Aleman A., Baune B.T., Berger K. (2021). Brain Aging in Major Depressive Disorder: Results from the ENIGMA Major Depressive Disorder Working Group. Mol. Psychiatry.

[17] Constantinides C., Han L.K.M., Alloza C., Antonucci L.A., Arango C., Ayesa-Arriola R., Banaj N., Bertolino A., Borgwardt S., Bruggemann J. (2023). Brain Ageing in Schizophrenia: Evidence from 26 International Cohorts via the ENIGMA Schizophrenia Consortium. Mol. Psychiatry.

[18] Beheshti I., Nugent S., Potvin O., Duchesne S. (2021). Disappearing Metabolic Youthfulness in the Cognitively Impaired Female Brain. Neurobiol. Aging.

[19] Franke K., Ziegler G., Klöppel S., Gaser C. (2010). Estimating the Age of Healthy Subjects from T1-Weighted MRI Scans Using Kernel Methods: Exploring the Influence of Various Parameters. NeuroImage.

[20] Beheshti I., Mishra S., Sone D., Khanna P., Matsuda H. (2020). T1-Weighted MRI-Driven Brain Age Estimation in Alzheimer’s Disease and Parkinson’s Disease. Aging Dis..

[21] Sone D., Beheshti I., Maikusa N., Ota M., Kimura Y., Sato N., Koepp M., Matsuda H. (2021). Neuroimaging-Based Brain-Age Prediction in Diverse Forms of Epilepsy: A Signature of Psychosis and Beyond. Mol. Psychiatry.

[22] Nenadić I., Dietzek M., Langbein K., Sauer H., Gaser C. (2017). BrainAGE Score Indicates Accelerated Brain Aging in Schizophrenia, but Not Bipolar Disorder. Psychiatry Res. Neuroimaging.

[23] Goyal M.S., Vlassenko A.G., Blazey T.M., Su Y., Couture L.E., Durbin T.J., Bateman R.J., Benzinger T.L.-S., Morris J.C., Raichle M.E. (2017). Loss of Brain Aerobic Glycolysis in Normal Human Aging. Cell Metab..

[24] Knopman D.S., Jack C.R., Wiste H.J., Lundt E.S., Weigand S.D., Vemuri P., Lowe V.J., Kantarci K., Gunter J.L., Senjem M.L. (2014). 18F-Fluorodeoxyglucose Positron Emission Tomography, Aging, and Apolipoprotein E Genotype in Cognitively Normal Persons. Neurobiol. Aging.

[25] Bonte S., Vandemaele P., Verleden S., Audenaert K., Deblaere K., Goethals I., Van Holen R. (2017). Healthy Brain Ageing Assessed with 18F-FDG PET and Age-Dependent Recovery Factors after Partial Volume Effect Correction. Eur. J. Nucl. Med. Mol. Imaging.

[26] Braak H., Thal D.R., Ghebremedhin E., Del Tredici K. (2011). Stages of the Pathologic Process in Alzheimer Disease: Age Categories from 1 to 100 Years. J. Neuropathol. Exp. Neurol..

[27] Lee J., Burkett B.J., Min H.-K., Senjem M.L., Lundt E.S., Botha H., Graff-Radford J., Barnard L.R., Gunter J.L., Schwarz C.G. (2022). Deep Learning-Based Brain Age Prediction in Normal Aging and Dementia. Nat. Aging.

[28] McKhann G.M., Knopman D.S., Chertkow H., Hyman B.T., Jack C.R., Kawas C.H., Klunk W.E., Koroshetz W.J., Manly J.J., Mayeux R. (2011). The Diagnosis of Dementia Due to Alzheimer’s Disease: Recommendations from the National Institute on Aging-Alzheimer’s Association Workgroups on Diagnostic Guidelines for Alzheimer’s Disease. Alzheimers Dement..

[29] Jack C.R., Wiste H.J., Weigand S.D., Therneau T.M., Lowe V.J., Knopman D.S., Gunter J.L., Senjem M.L., Jones D.T., Kantarci K. (2017). Defining Imaging Biomarker Cut Points for Brain Aging and Alzheimer’s Disease. Alzheimers Dement..

[30] MacMahon S., Peto R., Cutler J., Collins R., Sorlie P., Neaton J., Abbott R., Godwin J., Dyer A., Stamler J. (1990). Blood Pressure, Stroke, and Coronary Heart Disease. Part 1, Prolonged Differences in Blood Pressure: Prospective Observational Studies Corrected for the Regression Dilution Bias. Lancet.

[31] Bashyam V.M., Erus G., Doshi J., Habes M., Nasrallah I.M., Truelove-Hill M., Srinivasan D., Mamourian L., Pomponio R., Fan Y. (2020). MRI Signatures of Brain Age and Disease over the Lifespan Based on a Deep Brain Network and 14,468 Individuals Worldwide. Brain.

[32] Peng H., Gong W., Beckmann C.F., Vedaldi A., Smith S.M. (2021). Accurate Brain Age Prediction with Lightweight Deep Neural Networks. Med. Image Anal..

[33] Jonsson B.A., Bjornsdottir G., Thorgeirsson T.E., Ellingsen L.M., Walters G.B., Gudbjartsson D.F., Stefansson H., Stefansson K., Ulfarsson M.O. (2019). Brain Age Prediction Using Deep Learning Uncovers Associated Sequence Variants. Nat. Commun..

[34] Cole J.H., Franke K. (2017). Predicting Age Using Neuroimaging: Innovative Brain Ageing Biomarkers. Trends Neurosci..

[35] Benvenutto A., Giusiano B., Koric L., Gueriot C., Didic M., Felician O., Guye M., Guedj E., Ceccaldi M. (2018). Imaging Biomarkers of Neurodegeneration in Alzheimer’s Disease: Distinct Contributions of Cortical MRI Atrophy and FDG-PET Hypometabolism. J. Alzheimers Dis..

[36] Ossenkoppele R., Smith R., Ohlsson T., Strandberg O., Mattsson N., Insel P.S., Palmqvist S., Hansson O. (2019). Associations between Tau, Aβ, and Cortical Thickness with Cognition in Alzheimer Disease. Neurology.

[37] Wuestefeld A., Pichet Binette A., Berron D., Spotorno N., van Westen D., Stomrud E., Mattsson-Carlgren N., Strandberg O., Smith R., Palmqvist S. (2023). Age-Related and Amyloid-Beta-Independent Tau Deposition and Its Downstream Effects. Brain.

[38] Lee W.J., Brown J.A., Kim H.R., Joie R.L., Cho H., Lyoo C.H., Rabinovici G.D., Seong J.-K., Seeley W.W. (2022). Regional Aβ-Tau Interactions Promote Onset and Acceleration of Alzheimer’s Disease Tau Spreading. Neuron.

[39] Schoonhoven D.N., Coomans E.M., Millán A.P., van Nifterick A.M., Visser D., Ossenkoppele R., Tuncel H., van der Flier W.M., Golla S.S.V., Scheltens P. (2023). Tau Protein Spreads through Functionally Connected Neurons in Alzheimer’s Disease: A Combined MEG/PET Study. Brain.

[40] Millar P.R., Gordon B.A., Luckett P.H., Benzinger T.L., Cruchaga C., Fagan A.M., Hassenstab J.J., Perrin R.J., Schindler S.E., Allegri R.F. (2023). Multimodal Brain Age Estimates Relate to Alzheimer Disease Biomarkers and Cognition in Early Stages: A Cross-Sectional Observational Study. eLife.

[41] Biel D., Luan Y., Brendel M., Hager P., Dewenter A., Moscoso A., Otero Svaldi D., Higgins I.A., Pontecorvo M., Römer S. (2022). Combining Tau-PET and fMRI Meta-Analyses for Patient-Centered Prediction of Cognitive Decline in Alzheimer’s Disease. Alzheimers Res. Ther..

[42] Saint-Aubert L., Lemoine L., Chiotis K., Leuzy A., Rodriguez-Vieitez E., Nordberg A. (2017). Tau PET Imaging: Present and Future Directions. Mol. Neurodegener..

[43] Taylor A., Zhang F., Niu X., Heywood A., Stocks J., Feng G., Popuri K., Beg M.F., Wang L. (2022). Investigating the Temporal Pattern of Neuroimaging-Based Brain Age Estimation as a Biomarker for Alzheimer’s Disease Related Neurodegeneration. Neuroimage.

[44] Bejanin A., Schonhaut D.R., La Joie R., Kramer J.H., Baker S.L., Sosa N., Ayakta N., Cantwell A., Janabi M., Lauriola M. (2017). Tau Pathology and Neurodegeneration Contribute to Cognitive Impairment in Alzheimer’s Disease. Brain.

[45] Ossenkoppele R., van der Kant R., Hansson O. (2022). Tau Biomarkers in Alzheimer’s Disease: Towards Implementation in Clinical Practice and Trials. Lancet Neurol..

[46] Eavani H., Habes M., Satterthwaite T.D., An Y., Hsieh M.-K., Honnorat N., Erus G., Doshi J., Ferrucci L., Beason-Held L.L. (2018). Heterogeneity of Structural and Functional Imaging Patterns of Advanced Brain Aging Revealed via Machine Learning Methods. Neurobiol. Aging.

[47] Liu W., Dong Q., Sun S., Shen J., Qian K., Hu B. (2023). Risk Prediction of Alzheimer’s Disease Conversion in Mild Cognitive Impaired Population Based on Brain Age Estimation. IEEE Trans. Neural Syst. Rehabil. Eng..

[48] Cheng J., Liu Z., Guan H., Wu Z., Zhu H., Jiang J., Wen W., Tao D., Liu T. (2021). Brain Age Estimation From MRI Using Cascade Networks With Ranking Loss. IEEE Trans. Med. Imaging.

